# A comparison of statistical methods for genomic selection in a mice population

**DOI:** 10.1186/1471-2156-13-100

**Published:** 2012-11-08

**Authors:** Haroldo HR Neves, Roberto Carvalheiro, Sandra A Queiroz

**Affiliations:** 1 Departamento de Zootecnia, FCAV, UNESP, Jaboticabal, CEP: 14.884-900, SP, Brazil; 2 GenSys Consultores Associados S/S Ltda., Porto Alegre, Rio Grande do Sul, Brazil

**Keywords:** Kernel regression, LASSO, Random forest, Ridge regression, SNP, Subset selection

## Abstract

**Background:**

The availability of high-density panels of SNP markers has opened new perspectives for marker-assisted selection strategies, such that genotypes for these markers are used to predict the genetic merit of selection candidates. Because the number of markers is often much larger than the number of phenotypes, marker effect estimation is not a trivial task. The objective of this research was to compare the predictive performance of ten different statistical methods employed in genomic selection, by analyzing data from a heterogeneous stock mice population.

**Results:**

For the five traits analyzed (W6W: weight at six weeks, WGS: growth slope, BL: body length, %CD8+: percentage of CD8+ cells, CD4+/ CD8+: ratio between CD4+ and CD8+ cells), within-family predictions were more accurate than across-family predictions, although this superiority in accuracy varied markedly across traits. For within-family prediction, two kernel methods, Reproducing Kernel Hilbert Spaces Regression (RKHS) and Support Vector Regression (SVR), were the most accurate for W6W, while a polygenic model also had comparable performance. A form of ridge regression assuming that all markers contribute to the additive variance (RR_GBLUP) figured among the most accurate for WGS and BL, while two variable selection methods ( LASSO and Random Forest, RF) had the greatest predictive abilities for %CD8+ and CD4+/ CD8+. RF, RKHS, SVR and RR_GBLUP outperformed the remainder methods in terms of bias and inflation of predictions.

**Conclusions:**

Methods with large conceptual differences reached very similar predictive abilities and a clear re-ranking of methods was observed in function of the trait analyzed. Variable selection methods were more accurate than the remainder in the case of %CD8+ and CD4+/CD8+ and these traits are likely to be influenced by a smaller number of QTL than the remainder. Judged by their overall performance across traits and computational requirements, RR_GBLUP, RKHS and SVR are particularly appealing for application in genomic selection.

## Background

The availability of high-density panels of single nucleotide polymorphisms (SNP) containing thousands of markers opened new perspectives for the study of complex diseases, while has enhanced marker-assisted selection strategies in animal and plant breeding.

The possibility to predict accurately the genetic merit of selection candidates based on their genotypes for SNP markers, a process known as genomic selection [1], is revolutionizing breeding schemes. The reasoning of this process is that whenever marker density is high enough, most QTL will be in high linkage disequilibrium (LD) with some markers and estimates of marker effects will lead to accurate predictions of genetic merit for a trait.

Despite this, the amount of information to be analyzed in this situation poses new challenges from statistical and computational viewpoints. As the number of predictor variables (markers) is generally much higher than the number of observations (phenotypes), there is lack of degrees of freedom to estimate all marker effects simultaneously, what is aggravated by the fact that models may suffer from multicollinearity, especially because markers in close positions are expected to be highly correlated.

According to review in [2], some of the alternatives that have been employed to overcome these issues are fitting markers as random effects (e.g. shrinkage estimation and Bayesian regression) or applying some dimensionality reduction technique or machine learning method, although there is no consensus on the most appropriate method for genomic predictions. It has been argued that shrinkage methods with assumptions close to the infinitesimal model (i.e. GBLUP and its variants) are robust with respect to the underlying genetic architecture of the traits, while methods based on some sort of variable selection are more sensitive to the genetic background of traits [3,4].

There are still few extensive studies aimed to compare predictive performance of the such methods in plants or in animals [5]. In the present study, we analyze a publicly available dataset, including pedigree, genotypic and phenotypic information of a mice population. Although this same dataset had already been analyzed previously [6-8], we focus on a broader comparison of statistical methods employed for genomic prediction, by studying five traits that probably have considerable differences in terms of genetic architecture.

Thus, the objective of this research was to compare the predictive performance of ten different statistical methods employed in genomic selection by using data from a heterogeneous stock mice population, aiming to provide some insight in the scope of statistical methods useful for genomic selection and in the interplay between the genetic background of traits and the performance of these methods.

## Methods

### Data

The data came from a heterogeneous stock mice population kept by The Welcome Trust Centre for Human Genetics (WTCHG) (data are available at http://gscan.well.ox.ac.uk). Briefly, this population was generated from the crossing of eight inbred lines, followed by 50 generations of random mating. As a result, this population exhibits a high level of linkage disequilibrium, even for pairs of markers separated by until 2Mb [9]. When considering genotypic information obtained with a panel with 11,558 SNP markers and average inter-marker distance of 204 kb, the average r^2^ between adjacent markers was about 0.62 [6]. This amount of LD enhanced QTL mapping for complex traits in mice [10] and would be equally helpful in the context of genomic selection, besides the fact that knowledge of the origin of this population could improve interpretability of the results.

Only animals with both genotypes and phenotypes were considered and details of sampling and genotyping are described in Valdar et al. [11]. The raw data included genotypes for 12,226 SNP markers located in autosomes of 1,940 animals. Data were edited such that only polymorphic markers with MAF ≥ 5% and with no evidence of departure from Hardy-Weinberg equilibrium were considered in analyses.

Missing genotypes (0.1%) were imputed using probabilistic PCA (PPCA, [12]). Although the accuracy of this procedure is slightly lower than that of other methods, computing time is much lower. In addition, the proportion of missing genotypes is small enough to neglect the effects of imputation. After data editing, a dataset including information of 1,884 animals for 9,917 markers was considered in marker effect estimation, such that 168 full-sib families with average size of 11 were represented.

Five traits whose heritabilities are quite different were analyzed: percentage of CD8+ cells (%CD8+, h^2^=0.89), ratio between CD4+ and CD8+ cells (CD4+/ CD8+, h^2^=0.80), body weight at 6 weeks (W6W, h^2^ = 0.74), growth slope (WGS, h^2^=0.30), body length (BL, h^2^=0.13) [11]. Aiming to reduce computing times, phenotypes for each trait were pre-corrected for the significant environmental effects reported by [11].

Regarding to the genetic architecture of the traits in this study, an analysis of the supplementary material in [10] revealed that 17, 11, 19, 10 and 6 QTL were found to be significant on %CD8+, CD4+/ CD8+, W6W, GS and BL, respectively. For the first three of these traits, the QTLs mapped were responsible for more than 30% of the their variance (Table 1). The largest QTL with effects on %CD8+ and CD4+/ CD8+ explained about 8.0% and 12% of the variance of these traits, respectively, while the largest QTL on the other traits only accounted for about 3% or less of their variance.

**Table 1:** Available information* on the genetic architecture of the traits in study

**Trait**	**NºQTL**	**Variance explained (%)**	**Largest QTL (%)**	**Heritability**
%CD8+	17	36.3	8.00	0.89
CD4+/CD8+	11	33.1	11.90	0.80
W6W	19	38.3	3.20	0.74
WGS	10	20.6	2.40	0.30
BL	6	16.7	3.10	0.13

Trait = weight at 6 weeks (W6W), weight growth slope (WGS), body length (BL), percentage of CD8+ cells (%CD8+), ratio between CD4+ and CD8+ cells (CD4+/CD8+). Nº QTL = number of QTL mapped, proportion of the variance explained by them (variance explained, in %) and proportion of the variance explained by the QTL with largest effect(largest QTL, in %). ^*^Information published in Valdar et al. [10] and Valdar et al. [11].

When analyzing this dataset, Legarra et al. [6] alerted for the non-random allocation of animals between cages, in a way that many full-sib groups were kept in the same cage and thus additive and environmental effects were confounded at this level. For this reason, phenotypes were also adjusted for this random effect.

For each trait, REML estimates of variance components were obtained using all information available (pedigree and phenotypic records) and then phenotypes were adjusted for the environmental effects described previously.

### Study design

As our focus rely on the comparison of the performance of methods employed to estimate marker effects using real data, we employed a design similar to that employed by [6]. A cross-validation strategy was applied, such that data were split in two sets, reference (REF) and validation (VAL). For all methods, only the information on REF was employed to train the model, then solutions obtained in this step were used to predict the phenotypes of the animals in the VAL set.

The Pearson's correlation between phenotypes and their respective predictions $\left({\text{r}}_{y,\hat{y}}\right)$, hereinafter regarded as “predictive ability”, would allow comparison of predictive performance across methods. This approach has also a valuable interpretation in the context of animal breeding: the prediction of unobserved phenotypes mimics the prediction of the future performance of individuals in the population, as discussed in [6], in a way that the expected responses to selection using different methods could be compared.

Two-strategies for sampling animals were applied: (1) within-families, half-sib families were split such that about 55% (45%) of animals with phenotypes were included in the REF (VAL) set; (2) across-families, entire full-sib families were included in the REF set and used to predicted the observations of animals of other families (VAL set), such that REF set also comprised about 55% of the animals with phenotypes (Table 2). For each trait, ten replicates of each splitting strategy were done, such that empirical standard errors of parameters of interest were calculated based on nearly equal-sized partitions, ensuring that results were not due to random splitting of data. To ensure a more precise comparison, the different methods were applied in exactly the same partitions of the data.

**Table 2:** Summary statistics^*^ pertaining to phenotypic data^**^ employed in cross validation

**Trait**	**Split**	**N**	**Training size**			**Phenotypes training**		**Phenotypes testing**	
			**Min**	**Ave**	**Max**	**Mean**	**SD**	**Mean**	**SD**
W6W	within	1925	1059	1061.9	1066	−0.155	1.96	−0.188	1.95
WGS	within	1917	1056	1059.6	1068	0.001	0.04	0.002	0.04
BL	within	1840	1013	1017.9	1035	−0.002	0.40	−0.006	0.40
%CD8+	within	1407	774	778.6	785	0.010	4.34	0.122	4.36
CD4+/CD8+	within	1403	772	774.5	781	0.003	0.07	0.004	0.07
W6W	across	1925	1059	1067.6	1081	−0.162	1.95	−0.180	1.96
WGS	across	1917	1057	1063.3	1076	0.001	0.04	0.002	0.04
BL	across	1840	1014	1022.3	1030	−0.002	0.40	−0.005	0.40
%CD8+	across	1407	775	780.9	791	−0.149	4.23	0.319	4.43
CD4+/CD8+	across	1403	774	782.2	799	0.003	0.07	0.003	0.07

*N = total number of phenotypic records. Minimum, average and maximum size of training set (Min, Ave and Max) and mean and standard deviation (SD) of the adjusted records considered in training and testing sets (averaged across replicates).^**^Traits considered: weight at 6 weeks (W6W), weight growth slope (WGS), body length (BL), percentage of CD8+ cells (%CD8+), ratio between CD4+ and CD8+ cells (CD4+/CD8+). Split = splitting strategy in cross-validation (within or across-family).

It is important to emphasize that full-sib families considered in REF and VAL sets in this study are linked by distant relationships in the case of across-family splitting [6]. Thus, within-family predictions are expected to account for more recent relationships, while across-family predictions would mostly pick up LD persistent among families (i.e. older relationships).

### Genomic predictions

The following generic model was fitted to estimate the effect of markers on the trait Y:

(1) y=μ+Xg+e,

where y is the vector of adjusted phenotypes of order n, μ is an overall mean, X is a matrix of genotypes for p SNP loci (whose elements are indicator variables denoting number of copies of allele 1), g is a vector of SNP marker effects and e is a vector of random residual terms.

For all traits and sampling strategies, the following statistical procedures were employed to predict the phenotypes of the animals in VAL set:

- *RR_GBLUP*[1]: shrinkage method in which markers were treated as random effects, by solving mixed model equations defined in (1) considering the variance ratios calculated with REML estimates of residual variance (*σ*^2^_*e*_) and additive genetic variance (σ^2^_u_), obtained in a previous step.

Under these assumptions, the direct solution for equation (1) would be obtained as:

(2) $$\hat{g}={\left(X'X+\lambda I\right)}^{-1}\left(X'y\right)$$

where *λ* = σ^2^_e_/(*σ*^2^_u_/k), k=2 ∑ p_i_ − (1 − p_i_) and p_i_ is the allelic frequency of the i^th^ marker, as in [13], what reflects the fact that more polymorphic loci contribute more to the genetic variation.

In the present study, we employed an alternative method to solve (1) based on the SVD decomposition of X (i.e. X = UDV' = RV'), as proposed in [14]. These authors showed that identical solutions to those in (2) can be obtained by:

(3) $$\hat{g}=V{\left(R'R+\lambda I\right)}^{-1}\left(R'y\right)$$

what could be computationally advantageous when p >> n.

*emBayesB* : this procedure consists in a BayesB-like method implemented using the Expectation-Maximization algorithm proposed by [15]. A mixture distribution is assumed for marker effects - a proportion γ of them have effects drawn from a double exponential distribution, while the remainder effects are drawn from a Dirac Delta (DD) function, which has all its probability mass at 0. In the present study, the parameter γ was also estimated from the data.

- *SS_BY*: this method implemented a sort of subset selection through a two-step procedure. First step was carried out to select markers with significant effects on y through single-marker regression. The correction proposed by Benjamini & Yekutieli [16] was used to adjust p-values for multiple comparison (markers were selected using α = 1%). This procedure is often employed to control the false-discovery rate under dependence assumptions. In the second step, simultaneous estimation of the s selected markers was done similarly as in (2), by fitting them as random effects.

- *SS_ABS*: marker effects estimated with RR_GBLUP method were screened and those loci with larger contribution to the genetic variance (mean ± 1.5 SD) were selected. The variance at each locus was calculated as $2p_{i}\left(1-p_{i}\right){{\hat{g}}_{i}}^{2}$, where p_i_ is the allelic frequency and ĝ the estimated effect for the i^th^ locus. In the second step, simultaneous estimation of the selected markers was done similarly as in (2).

*RKHS*: Reproducing Kernel Hilbert Spaces regression using a Gaussian kernel was carried out by fitting the following model:

$$y=\mu +K_{h}\alpha +e\text{,}$$

under the assumption of the following prior distributions *α* ∼ N(0, K_h_*σ*_*α*_^2^) and e ∼ N(0, I*σ*_*e*_^2^). The entries of the kernel matrix K_h_ were defined as:

$$K_{h}\left(x_{i},x_{j}\right)=\exp\left(-hd_{\mathrm{ij}}\right)\text{,}$$

where the d_ij_ the squared Euclidean distance between individuals i and j calculated based on their genotypes for SNP markers and the smoothing parameter h was defined as h = 2/d^*^ and d^*^ is the mean of d_ij_. This method was implemented in a Bayesian framework by using a Gibbs sampler, similarly as described by [17].

*SVR*: Support vector regression was implemented using a radial basis kernel. Briefly, this method employs linear models to map (implicitly) the data to a higher-dimensional space via a kernel function. As discussed in [18], one feature of this method is to minimize a cost function that simultaneously includes model complexity and error in the training data. The regularization parameter was set to 1 as well as the default values of the tuning parameters of the function svm (R package 'e1071') were adopted.

-*BayesCpi*: By following notation from the equation (1), this method postulates a mixture model for marker effects such that the elements of vector Xg were calculated for each animal as $\sum_{j=1}^{N}\left(x_{j}a_{j}I_{j}\right)$, where *x*_*j*_ is the genotype of the j^th^ marker, coded as the number of copies of one allele, *a*_*j*_ is the effect of marker j and *I*_*j*_ is an indicator variable that assumes the value of 1 whether the j^th^ marker has any effect on the trait or 0, otherwise.

It was assumed that *a*_*j*_ ∼ N(0, σ^2^_a_) and *e* ∼ N(0, Iσ^2^_e_). Inverted scaled chi-squared distributions were postulated for σ^2^_*a*_ and σ^2^_e_ as described in [19]. A binomial distribution with probability (1-π) was assumed for *I*_*j*_ and an uniform prior was assigned for π. This model was implemented using a Gibbs sampler, such that a single chain of 50,000 iterations was simulated, the first 5,000 being discarded as burn-in.

Note that, unlike in BayesB method [1], this mixture model assumes that marker effects are sampled from the same (normal) distribution, instead of estimating marker-specific variances.

-*BayesC*: a similar model to that described for Bayes Cpi was fitted, differing of that by the fact that the parameter π was kept fixed at 0.90.

- *LASSO*[20]: this method can be understood as a shrunken version of least squares estimates, obtained after minimizing the residual sum of squares subject to the restriction that L1-norm of ĝ (i.e. sum of the absolute value of marker effects ) must be ≤ t. The threshold t was defined by means of internal cross-validation (10-fold).

*RF*: the Random Forest algorithm [21] was applied in a regression framework, by assuming the matrix X as predictor of the phenotypes in y. A random forest of 1000 trees was built and this model was used to predict observations of VAL set.

### Implementation

All analyses were performed using the R software [22]. In order to avoid the direct inversion of large matrices, the GSRU algorithm [23] was employed to solve iteratively the linear systems in GBLUP, SS_BY*,* SS_ABS and emBayesB. To speed up computations, the implementations for GBLUP, SS_BY, SS_ABS, emBayesB, BayesCpi and BayesC were compiled in C++ language, by using Rcpp package. The method RKHS was implemented using the R code provided by [17]. The other methods were implemented using specific R packages: e1071 (SVR), glmnet (LASSO) and randomForest (RF). REML estimates of variance components were obtained using ASREML-R package [24]. All the analyses were performed on a workstation with a Intel i7-2600 3.40GHz processor and 8GB RAM.

### Analyses of results

All methods were compared based on their predictive ability $\left({\text{r}}_{\text{y,}\hat{\text{y}}}\right)$, calculated as the Pearson's correlation between the phenotypes of each animal in the VAL set and the respective predicted values $\left(\hat{\text{y}}\right)$. This statistic was also computed for a situation in which only information of pedigree and phenotypes was considered (polygenic model, POL), such that gains in predictive ability due to the consideration of genotypic information could be evaluated. For POL, the predicted values of observations were EBVs of VAL animals, obtained when considering exclusively the phenotypic information on animals in the REF set.

Significant differences between methods in terms of predictive ability were assessed by means of paired t tests (α = 5%), adjusted by Bonferroni correction.

The bias of prediction of each method was measured by the average prediction error, while the trend of inflation was measured by the slope of the regression of the observed phenotypes (y) on their predicted values $\left(\hat{\text{y}}\right)$. Mean squared error (MSE) was employed as a measure of the overall fit achieved with each method. As a general rule, values for bias (inflation) close to zero (close to 1) indicate better performance. As the phenotypes for each trait are in different scales, MSE was normalized (NRMSE). NRMSE was computed as the root mean-squared error divided by the range of the observed values. Values close to zero for NRMSE are associated with better overall fit.

Averages and standard errors (SE) were computed for each statistic by considering the results of the ten replicates available in each situation. The computing times required for the implementation of each method were also monitored and compared.

In the case of the methods which explicitly estimate marker effects, the distributions of marker effects were also examined and compared.

The accuracy of RR_GBLUP was calculated as its predictive ability divided by the square-root of the heritability of each trait [25] and then compared with the expected value for this statistic $\left(r_{g,\hat{g}}\right)$, derived according to the formula in Daetwyler et al. (2010):

$$r\left(g,\hat{g}\right)=\sqrt{\left(\frac{Nh^{2}}{Nh^{2}+Me}\right)}\text{,}$$

where N is the (average) size of the reference set, h^2^ is the (pseudo)heritability of the trait and Me is the number of independent chromosome segments, calculated as *Me = 2NeL/ln(4NeL)* or *Me = 2NeL*[26]. *L* is the length of the genome in Morgans and Ne is the effective population size (calculated in present study based on the estimates of r^2^ between SNP markers). The values of h^2^ considered in the formula accounted for the fact that phenotypes were adjusted for the effect of cage.

### Variation in accuracy across genetic groups

Heslot et al. [5] verified that large differences in accuracy between subpopulations could not be explained only by differences in phenotypic variance and sample size. Although the definition of subpopulations is not so obvious in present study, it would be reasonable to investigate differences in accuracy of prediction between the unrelated families comprising the mice dataset. Because family sizes are not large enough to enable calculation of predictive ability within each of such families, we investigated this question by clustering the individuals into groups according to the genetic distance between them.

For this, a hierarchical clustering algorithm (Ward's method) was applied to a matrix of genetic distances calculated based on the genomic relationship matrix between the animals, in order to identify non-trivial partitions of the data. The Calinski-Harabaz statistic was employed to find the optimal number of clusters and after this procedure, the solution obtained with Ward's method was refined using k-means algorithm.

For both within-family and across-family splitting, predictive abilities were calculated within each one of the genetic groups obtained through clustering, for each combination of method, trait and replicate. A Fligner-Killeen test was applied to assess homogeneity of phenotypic variances across groups, such that we could investigate whether eventual differences in predictive ability between groups could be related to differences in phenotypic variances.

In order to test for differences in within-group predictive ability, a Fisher's z transformation was applied over predictive abilities, since these are computed as Pearson's correlations and thus their sampling distributions are not normal. Then, for each replicate, equality of predictive ability across groups was assessed using a chi-square test, after which p-values were averaged across replicates.

## Results

### Variance components

REML estimates of variance components are presented for each trait in Table 3. Estimated heritabilities for W6W, WGS, BL, %CD8+ and CD4+/CD8+ matched well the previous estimates published by Valdar et al. [11] and presented in Table 1. Also, the estimates for W6W, WGS and BL were in agreement to those obtained by [6], being that the largest difference was observed for body length, whose heritability was 7% lower in the present study.

**Table 3:** REML estimates of variance components (and related parameters) for traits of a heterogeneous stock mice population

**Trait**	**σ^2^_u_**	**SE**	**σ^2^_c_**	**SE**	**σ^2^_e_**	**SE**	**h^2^**	**SE**
W6W	3.915	29.836	1.719	13.100	3.E-05	9.E-04	0.695	0.030
WGS	8.E-04	2.E-04	1.E-03	1.E-04	9.E-04	1.E-04	0.295	0.069
BL	0.036	0.012	0.039	0.007	0.148	0.009	0.161	0.051
%CD8+	19.370	2.851	1.990	0.471	0.357	1.505	0.892	0.101
CD4+/CD8+	5.E-03	7.E-04	6.E-04	1.E-04	4.E-04	4.E-04	0.825	0.081

Trait = weight at 6 weeks (W6W), weight growth slope (WGS), body length (BL), percentage of CD8+ cells (%CD8+), ratio between CD4+ and CD8+ cells (CD4+/CD8+).

σ^2^_u_: additive genetic variance ; σ^2^_c_: variance due the random environmental effect of cage; σ^2^_e_: residual variance; h^2^: heritability (standard error, SE, in brackets).

### Within-family predictions

**Figure 1:**
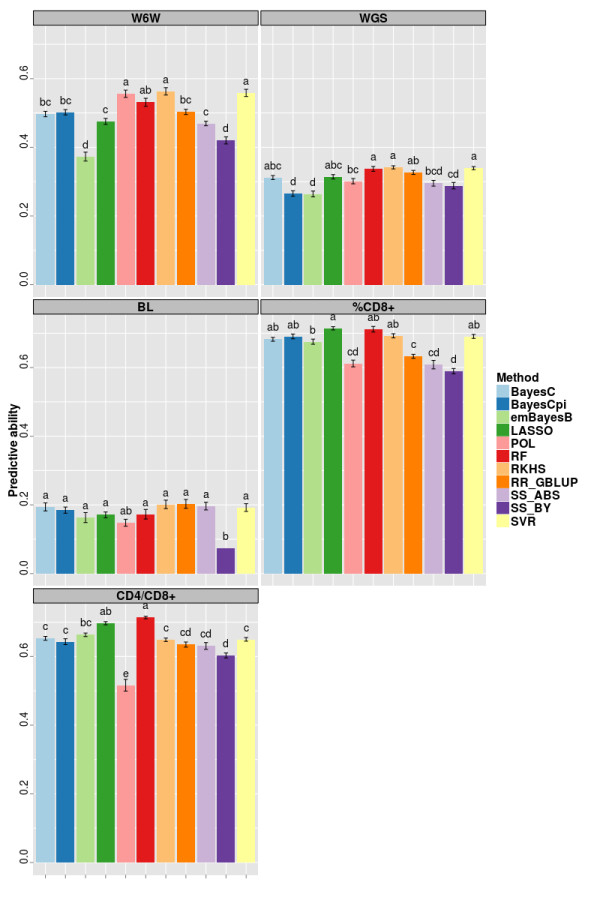
Predictive ability* of the different methods employed in within-family predictions for five traits in a mice population. *Average of ten replicates. Bars sharing the same letter are not different (P >0.05). Traits: weight at 6 weeks (W6W), weight growth slope (WGS), body length (BL), percentage of CD8+ cells (%CD8+), ratio between CD4+ and CD8+ cells (CD4+/CD8+).

In Figure 1, results of predictive ability under within-family splitting are presented for all methods, grouped by trait, as well as the results obtained when considering only pedigree and phenotypic information (i.e. using the polygenic model, POL). The polygenic model achieved predictive abilities about 0.56, 0.30, 0.15, 0.61 and 0.52 for W6W, WGS, BL, %CD8+ and CD4+/ CD8+, respectively.

For a same trait, some methods had comparable predictive abilities, although significant differences between methods could be found. For all traits, at least two of the methods (SVR and RKHS) reached greater predictive abilities than POL (Figure 1).

Also, the relative performance of the methods varied noticeably across traits. RKHS, POL and SVR (in this order) were the most accurate for W6W, while RKHS, SVR and RF outperformed the remainder methods with respect to the predictions for WGS. Predictions for BL did not differ greatly across methods, except by the worst performance of SS_BY. LASSO and RF were the two with greater predictive abilities for %CD8+ and CD4+/ CD8+. As a general rule, the methods based in some sort of variable selection (especially LASSO and emBayesB) had better performance in the case %CD8+ and CD4+/ CD8+ compared to the other traits.

Overall, the subset selection methods (SS_ABS and SS_BY) did not rank among the best methods for none of the traits studied. Also, it is important to mention that for BL, the significance threshold applied in SS_BY was possibly too stringent, since that in only one of the ten replicates significant markers were found, reason why error bars for predictive ability and fitting statistics are not presented in this situation.

Because methods with assumptions close to RR_GBLUP are among the most used in practical applications of genomic selection, it is meaningful to assess the additional gain in predictive ability that can be reached by methods with different assumptions. In present study, predictive ability of RR_GBLUP figured among the highest in the case of predictions for BL and WGS. For the remainder traits, the most accurate methods reached predictive abilities between 12% and 13% greater than RR_GBLUP.

An additional set of analyses was carried out by considering a smaller MAF threshold (1%) for genotypes, aiming to investigate whether lower frequency variants could be important for some of the traits under investigation. As a general rule, predictive abilities of the two sets of analyses did not differ by more than 0.5%, being that the largest increase (2.9%) was observed for WGS when using BayesCpi (data not shown).

### Across-family predictions

It must be noted that across-family predictions using method POL are expected to have accuracy of zero, because the pedigree information do not include links between animals in REF and VAL sets, although the predictive ability cannot be explicitly computed in this case, since the SD of the predicted values for VAL set is zero.

**Figure 2:**
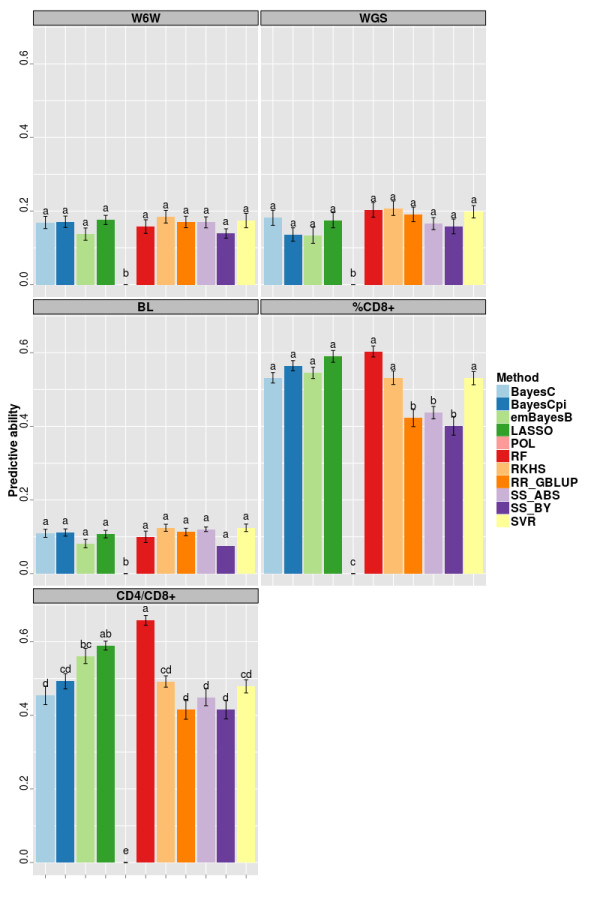
Predictive ability* of the different methods employed in across-family predictions for five traits in a mice population. Average of ten replicates. Bars sharing the same letter are not different (P >0.05). Traits: weight at 6 weeks (W6W), weight growth slope (WGS), body length (BL), percentage of CD8+ cells (%CD8+), ratio between CD4+ and CD8+ cells (CD4+/CD8+).

For the remainder methods, predictive ability was consistently lower in across-family predictions (Figure 2) compared to within-family predictions (Figure 1). Across methods, the greatest decreases in predictive ability relative to within-family predictions were observed for W6W(66%), WGS (44%) and BL (41%), for which predictive abilities reached figures about 0.20 at most and no significant differences between methods were found.

For %CD8+ and CD4+/ CD8+, predictive ability averaged across methods was about 0.50 and thus about 22% lower than in within-family splitting. RF and LASSO (in this order) had the highest predictive abilities for both traits (Figure 2), although other methods reached comparable predictive ability for %CD8+. For these traits, the advantage over RR_GBLUP was more pronounced when compared to the results obtained for within-family predictions. For example, the most accurate method (RF) reached predictive abilities about 43% (%CD8+ ) and 58% (CD4+/ CD8+) greater than RR_GBLUP (Figure 2).

### Bias, inflation and overall fit

Since the phenotypes for each trait are in different scales, the averages of bias are presented as proportions of the respective phenotypic SD in Table 4. Except for W6W and regardless of the splitting strategy, the largest amount of bias were observed for BayesC and BayesCpi, for which there was a trend of overestimation in the case WGS and BL, while the predictions for the other traits were underestimated. Predictions for LASSO and emBayesB were considerably underestimated in the case of W6W, %CD8+ and CD4+/ CD8+ and overestimated for WGS (Table 4). Overall, the less biased predictions were obtained with RF, RKHS, SVR and RR_GBLUP.

**Table 4:** Bias^*^ of genomic predictions from different methods, obtained for five traits of a mice population

**Splitting**	**Method**	**Trait**				
		**W6W**	**WGS**	**BL**	**%CD8+**	**CD4+/CD8+**
Within	POL	−4%	1%	−1%	1%	1%
	emBayesB	−62%	24%	2%	−42%	−52%
	RR_GBLUP	−9%	8%	13%	2%	4%
	SS_BY	−18%	22%	-	5%	5%
	SS_ABS	−35%	4%	−30%	5%	17%
	RKHS	−1%	1%	−1%	1%	2%
	SVR	−4%	3%	−6%	5%	4%
	BayesCpi	−26%	66%	81%	−568%	−91%
	BayesC	−43%	263%	69%	−397%	−420%
	LASSO	−85%	53%	2%	−73%	−73%
	RF	−1%	1%	1%	0%	1%
Across	POL	-	-	-	-	-
	emBayesB	−51%	5%	3%	−58%	−87%
	RR_GBLUP	−31%	8%	8%	−2%	14%
	SS_BY	−44%	18%	-	−1%	17%
	SS_ABS	−54%	−7%	−38%	−16%	31%
	RKHS	−23%	4%	−4%	9%	10%
	SVR	−24%	6%	−8%	14%	10%
	BayesCpi	−15%	83%	46%	−719%	−196%
	BayesC	23%	280%	115%	−239%	−359%
	LASSO	−116%	40%	7%	−70%	−98%
	RF	−13%	2%	−1%	5%	5%

*Average of ten replicates. Bias was measured as the average difference between observed and predicted phenotypes of testing set and is presented as a proportion of the standard deviation of each trait (in %). Trait = weight at 6 weeks (W6W), weight growth slope (WGS), body length (BL), percentage of CD8+ cells (%CD8+), ratio between CD4+ and CD8+ cells (CD4+/CD8+). Splitting= splitting strategy in cross-validation (within or across-family).

The methods under investigation also differed greatly in terms of the inflation of genomic predictions, being that BayesCpi and BayesC were those producing the most inflated genomic predictions for all traits, followed by SS_BY and SS_ABS, while emBayesB was the only method which consistently resulted in deflation of genomic predictions, under within-family splitting (Table 5). Across traits, LASSO and RKHS had the coefficients of inflation closest to 1 for within-family predictions, followed by SVR, RF and RR_GBLUP. In most of the situations, the coefficients of inflation for across-family predictions showed greater deviation from 1.

**Table 5:** Inflation^*^ of genomic predictions from different methods, obtained for five traits of a mice population

**Splitting**	**Method**	**Trait**				
		**W6W**	**WGS**	**BL**	**%CD8+**	**CD4+/CD8+**
Within	POL	1.21	0.91	0.89	1.03	0.97
	emBayesB	1.43	1.57	3.70	1.65	1.55
	RR_GBLUP	0.95	0.70	0.88	0.66	0.75
	SS_BY	0.79	0.66	-	0.60	0.72
	SS_ABS	0.72	0.49	0.49	0.64	0.72
	RKHS	1.08	0.95	0.89	1.06	1.26
	SVR	1.24	0.91	0.68	1.16	1.18
	BayesCpi	0.55	0.29	0.40	0.17	0.29
	BayesC	0.18	0.12	0.21	0.19	0.20
	LASSO	0.93	1.01	1.08	1.00	1.07
	RF	1.48	1.10	0.78	1.12	1.11
Across	POL	-	-	-	-	-
	emBayesB	0.46	0.90	1.47	1.43	1.33
	RR_GBLUP	0.37	0.49	0.52	0.46	0.52
	SS_BY	0.30	0.46	-	0.42	0.52
	SS_ABS	0.29	0.30	0.32	0.50	0.52
	RKHS	0.55	0.95	0.75	1.21	1.36
	SVR	0.61	0.89	0.62	1.42	1.27
	BayesCpi	0.22	0.21	0.14	0.17	0.24
	BayesC	0.07	0.08	0.11	0.18	0.15
	LASSO	0.38	0.71	0.56	0.98	1.01
	RF	0.72	1.22	0.65	1.27	1.22

^*^Average of ten replicates. Inflation was measured as the slope of the regression of observed phenotypes on predicted phenotypes of testing set

Trait = weight at 6 weeks (W6W), weight growth slope (WGS), body length (BL), percentage of CD8+ cells (%CD8+), ratio between CD4+ and CD8+ cells (CD4+/CD8+). Splitting= splitting strategy in cross-validation (within or across-family).

In terms of overall fit, measured by the normalized root-mean squared error, BayesC and BayesCpi were again those with worst performance, what is expected judged by their performance in terms inflation and mainly bias, which are accounted for by MSE (Table 6). Also, by averaging NRMSE across methods, it can be noted that predictions for %CD8+ and CD4+/ CD8+ showed greater values for NRMSE than those of the other traits.

**Table 6:** Normalized root-mean squared error(NRMSE)^*^ of genomic predictions from different methods, obtained for five traits of a mice population

**Splitting**	**Method**	**Trait**				
		**W6W**	**WGS**	**BL**	**%CD8+**	**CD4+/CD8+**
Within	POL	0.099	0.114	0.123	0.138	0.145
	emBayesB	0.140	0.122	0.124	0.165	0.165
	RR_GBLUP	0.103	0.114	0.123	0.147	0.136
	SS_BY	0.112	0.122	-	0.158	0.144
	SS_ABS	0.117	0.120	0.132	0.161	0.143
	RKHS	0.098	0.112	0.122	0.127	0.131
	SVR	0.099	0.112	0.123	0.128	0.130
	BayesCpi	0.166	0.214	0.268	1.252	0.502
	BayesC	0.534	0.877	0.251	1.035	1.080
	LASSO	0.172	0.137	0.127	0.204	0.196
	RF	0.102	0.112	0.123	0.124	0.119
Across	POL	-	-	-	-	-
	emBayesB	0.154	0.132	0.126	0.199	0.230
	RR_GBLUP	0.128	0.121	0.125	0.184	0.170
	SS_BY	0.141	0.126	-	0.193	0.175
	SS_ABS	0.145	0.130	0.140	0.188	0.180
	RKHS	0.122	0.119	0.124	0.152	0.152
	SVR	0.122	0.119	0.125	0.155	0.153
	BayesCpi	0.163	0.243	0.240	1.519	0.593
	BayesC	0.637	0.747	0.275	0.896	1.091
	LASSO	0.201	0.142	0.129	0.213	0.225
	RF	0.119	0.119	0.124	0.144	0.130

*Average of ten replicates. Lower values are associated with better overall fit

Trait = weight at 6 weeks (W6W), weight growth slope (WGS), body length (BL), percentage of CD8+ cells (%CD8+), ratio between CD4+ and CD8+ cells (CD4+/CD8+). Splitting= splitting strategy in cross-validation (within or across-family).

By considering the overall fit (Table 6), it can be noted a more consistent ranking of methods when compared to the results for predictive ability (Figures 1 and 2). In the case of within-family predictions, RKHS was the best method for W6W, WGS and BL, while RF was the best for the other two traits. Typically, RF, RKHS and SVR were the best three methods in terms of overall fit what was also observed in the case of across-family predictions.

### Distribution of marker effects

The variation of the excess kurtosis of the distribution of the marker effects estimated for different traits could indicate that a method is able to fit marker effect distribution to the QTL distributions of such traits. Results for this statistic are presented in Table 7. As a general rule, although the magnitude of excess kurtosis of effect distribution differed greatly among methods for a same trait, estimates of this statistic were reasonably consistent for a same method. The only exception was found in the case of BayesCpi, for which the marker effect distribution was close to a normal distribution for W6W, while more peaked distributions were found for the other traits. For the sake of brevity, results for across-family splitting are not presented, as the findings were very similar to those observed under within-family splitting.

**Table 7:** Summary statistics* associated with distributions of estimated marker effects (within-family splitting)

		**Method**					
**Trait**	Stat*	**BayesCpi**	**emBayesB**	**RR_GBLUP**	**LASSO**	**SS_ABS**	**SS_BY**
BL	t1000	0.83	1.00	0.52	1.00	1.00	1.00
	t500	0.75	1.00	0.35	1.00	0.99	0.97
	t100	0.53	0.99	0.12	0.97	0.52	0.76
	t20	0.34	0.96	0.04	0.68	0.18	0.43
	|g| > 0	9457	4076	9820	199	623	541
	kurt	284.0	2650.1	0.6	496.3	37.7	503.7
CD4+/CD8+	t1000	1.00	1.00	0.53	1.00	1.00	0.96
	t500	0.99	1.00	0.37	1.00	0.99	0.88
	t100	0.92	0.99	0.13	0.97	0.57	0.60
	t20	0.64	0.97	0.04	0.71	0.21	0.32
	|g| > 0	9559	3938	9820	228	577	1172
	kurt	407.2	2790.4	1.0	597.7	48.6	319.9
%CD8+	t1000	0.91	1.00	0.53	1.00	1.00	0.98
	t500	0.83	1.00	0.36	1.00	0.99	0.93
	t100	0.50	0.99	0.13	0.97	0.54	0.69
	t20	0.21	0.96	0.04	0.71	0.19	0.39
	|g| > 0	9820	3792	9820	203	603	754
	kurt	36.1	3009.2	0.7	553.6	41.2	443.6
W6W	t1000	0.51	1.00	0.52	1.00	1.00	1.00
	t500	0.35	1.00	0.36	1.00	0.99	1.00
	t100	0.12	1.00	0.12	0.93	0.54	0.85
	t20	0.04	1.00	0.04	0.49	0.19	0.46
	|g| > 0	9820	4711	9820	325	612	456
	kurt	0.7	1094.8	0.6	218.0	39.9	155.5
WGS	t1000	1.00	1.00	0.52	1.00	1.00	1.00
	t500	1.00	1.00	0.35	1.00	0.99	0.98
	t100	1.00	1.00	0.12	0.95	0.54	0.77
	t20	0.93	1.00	0.04	0.58	0.19	0.40
	|g| > 0	1716	4623	9820	260	617	655
	kurt	877.0	1998.6	0.6	310.9	40.1	134.7

*Average of 10 replicates. t1000, t500, t100 and t20 = proportion of the variance accounted for the markers (varM) explained by those with the largest 1000, 500, 100 and 20 absolute effects, respectively. $\mathrm{var}\text{M=}\sum_{j}\left[{{\hat{g}}_{i}}^{2}2p_{i}\left(1-p_{i}\right)\right]$, in which ${\hat{g}}_{i}$ and p_i_ are the estimated effect and the allele frequency for the ith marker, respectively. |*g*| > 0 = number of markers with non-null estimated effect. kurt = excess kurtosis of the distribution of estimated marker effects. Trait = weight at 6 weeks (W6W), weight growth slope (WGS), body length (BL), percentage of CD8+ cells (%CD8+), ratio between CD4+ and CD8+ cells (CD4+/CD8+).

A further inspection on the distribution of estimated marker effects, showed that, for the variable selection methods, a given proportion of the markers contributed a smaller proportion of the total genetic variance accounted by the markers in W6W compared to the other traits (Table 7). The estimates of the proportion of markers with effect on each trait obtained using BayesCpi, by averaging the posterior means of (1-π) across replicates, were 59.0% (W6W), 0.1% (WGS), 11% (BL), 2.1% (%CD8+) and 0.4% (CD4 +/ CD8+) and also suggested a more polygenic control on W6W.

### Computing time

**Figure 3:**
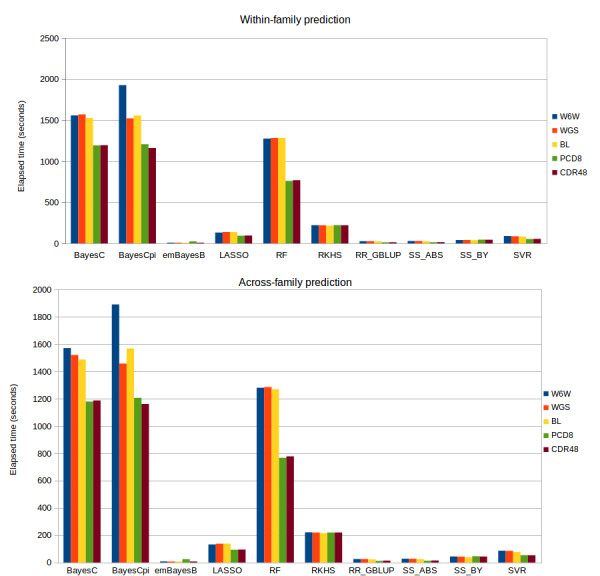
Average computing time* required to perform model training using different statistical methods. *Average of ten replicates. Elapsed times were measured during model training, carried out with the information available in the reference set, aiming to compute genomic predictions for five traits in a mice population.

The elapsed time to perform model training was of order of minutes for all methods investigated and the ranking of the methods for this criterion was consistent across traits. The more demanding methods were BayesCpi, BayesC and RF (in this order), whose computing times were between 5-fold and 131-fold larger than those required by the other methods (Figure 3). The two lowest computing times were measured for emBayesB and RR_GBLUP.

### Expected and realized accuracy of RR_GBLUP

**Figure 4:**
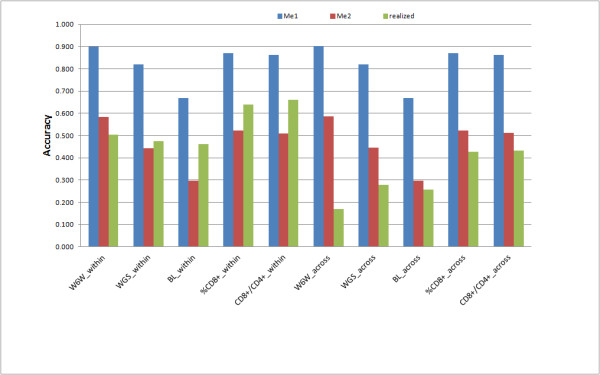
**Expected and realized accuracy of genomic predictions with RR_GBLUP.** Expected accuracies were calculated according to Daetwyler et al. (2010), by considering two approximations for the number of independent chromosome segments (Me): Me1 = 2NeL/ln(4NeL) or Me2 = 2NeL, where Ne is the effective population size and L is the genome length (in Morgans). realized = realized accuracy of GBLUP (average of ten replicates). Five traits were considered: weight at 6 weeks (W6W), weight growth slope (WGS), body length (BL), percentage of CD8+ cells (%CD8+), ratio between CD4+ and CD8+ cells (CD4+/CD8+). Two scenarios of prediction were considered within-family (_within) and across-family predictions (_across).

In Figure 4, expected accuracies of RR_GBLUP are presented for the two approximations of the number of independent chromosome segments (Me1 and Me2), besides the realized values obtained in each situation. It can be seen that the way Me was approximated impacted heavily on expected accuracies. As a general rule, the realized accuracies of RR_GBLUP matched better the expected values in the case of within-family predictions and the expected values computed assuming *Me=2NeL* fitted best to the realized values.

### Variation in predictive ability across genetic groups

According to the Calinski-Harabaz statistic, the optimal number of groups was found to be four (data not shown). There was evidence of differences between these roups for phenotypic variance in the case of %CD8+ (P=0.00031), CD4+/CD8+ (P=0.00034) and WGS (P=0.04137), while significant differences were not found for the other traits (P>0.20).

Although the chi-square test for equality of within-group predictive abilities takes in account differences in sample size, there were remarkable differences in the p-values obtained across replicates for a same trait and method, such that, when differences were averaged, they did not provide strong evidence against the null hypothesis in most of the situations analyzed (Table 8).

**Table 8:** Summary of the results^*^ of the test for equality of predictive ability across groups

**Splitting**	**Within-family**					**Across-family**				
**Trait**	**W6W**	**WGS**	**BL**	**%CD8+**	**CD4+/ CD8+**	**W6W**	**WGS**	**BL**	**%CD8+**	**CD4+/ CD8+**
emBayesB	0.42	0.30	0.26	0.28	0.05	0.22	0.47	0.33	0.06	0.03
RR_GBLUP	0.35	0.41	0.27	0.19	0.11	0.30	0.36	0.28	0.10	0.09
SS_BY	0.32	0.36	0.16	0.13	0.07	0.40	0.30	0.20	0.09	0.03
SS_ABS	0.39	0.34	0.28	0.04	0.17	0.20	0.28	0.34	0.06	0.06
RKHS	0.38	0.33	0.33	0.24	0.06	0.30	0.41	0.28	0.28	0.06
SVR	0.41	0.34	0.33	0.25	0.08	0.28	0.39	0.35	0.30	0.05
BayesCpi	0.37	0.28	0.27	0.28	0.07	0.31	0.38	0.25	0.12	0.07
BayesC	0.34	0.44	0.25	0.27	0.08	0.29	0.37	0.26	0.19	0.06
LASSO	0.45	0.29	0.24	0.24	0.10	0.30	0.36	0.38	0.10	0.03
RF	0.40	0.35	0.32	0.16	0.07	0.29	0.62	0.27	0.16	0.13

*P-values for the chi-square test for equality of predictive ability across groups (average of 10 replicates). In order to reduce the influence of discrepant replicates, the average of log10(p-value) was calculated for each trait and method, and then back-transformed to the original scale. Animals were clustered into 4 groups based on genetic distance between them.

Trait = weight at 6 weeks (W6W), weight growth slope (WGS), body length (BL), percentage of CD8+ cells (%CD8+), ratio between CD4+ and CD8+ cells (CD4+/CD8+). Splitting= splitting strategy in cross-validation (within or across-family).

Despite this, significant results (or at least suggestive) of differences in predictive abilities between groups were found in the case of CD4+/CD8+ and %CD8+, being that the magnitude of such differences also varied across methods. As a general rule, for these traits, stronger evidence for differences between groups in predictive ability was found under across-family splitting compared to within-family splitting.

## Discussion

### Previous studies with the mice dataset

As mentioned earlier, the mice dataset was also object of previous studies. [6] and [8] analyzed W6W, WGS and BL, using methods that are analogous to RR_GBLUP and LASSO, respectively, while [7] analyzed %CD8+ through a Bayesian variable selection method (RJMCMC) and thus somewhat analogous to the variable selection methods of this study (especially BayesCpi). Despite this, it is important to mention that results of these different studies are not fully comparable, due to the influence of data editing and random splitting procedures and even due to differences in method implementation.

For within-family predictions, as a general rule, predictive abilities were between 19% and 40% lower in the present study than in comparable situations reported by [6] and [8] what can be attributed mainly to differences in the way the cage effects were modeled. In present study, the phenotypes were adjusted for cage effects, while in that studies such effects were fitted simultaneously to marker effects and thus contributed to predictive ability. An additional argument in favor of this hypothesis is that similar differences were also observed when comparing model POL to an analogous model fitted by [6].

On the other hand, for %CD8+, emBayesB, BayesCpi and LASSO achieved predictive abilities 4%, 6% and 10% greater, respectively, than that obtained with RJMCMC under within-family prediction [7]. These authors also verified that, when dominance effects on this trait were fitted simultaneously to additive effects, predictive ability increased by about 6% and 10% for within-family and across-family prediction, respectively.

### Comparison between statistical methods in genomic prediction

While simulation studies suggest that variable selection methods (e.g. BayesB, LASSO) could outperform methods with assumptions close to those of GBLUP (e.g. [1,4,27]), the performance of GBLUP has often been comparable to that of variable selection methods when real data were analyzed (e.g. [18,28,5]) . A possible explanation for the apparent divergence of results with simulated and real data could be that, for real data, the distribution of QTL effects for most traits is less extreme than has been simulated, as suggested by [3] and [29].

In the present study, methods with large conceptual differences reached very similar predictive abilities in some situations and a clear re-ranking of methods was observed in function of the trait analyzed. In other species, it has also been verified that methods with different assumptions had similar performance (e.g. [28,30,5]), while method by trait interaction often takes place (e.g. [31,32]).

Overall, for at least one of the five traits analyzed in present study, most of the methods figured among the most accurate, being that the only exception was observed for SS_BY and SS_ABS, reason why these methods do not seem to be recommendable for application in genomic selection.

One important point that needs to be taken into account when comparing methods employed in genomic selection is that their performance is often influenced by key parameters required in their implementations, like the assignment of prior distributions in Bayesian regression methods and setting tuning parameters of machine learning methods. In this way, such parameters can be optimized for each situation analyzed in order to improve predictive performance.

Although we expected that the implementations of the LASSO (tuned through internal cross-validation) and emBayes (in which parameters related to the distribution of marker effects are estimated from the data) also could fit well to QTL distribution, results of excess kurtosis suggest that statistical learning was more effective in the case of BayesCpi, although some drawbacks were found for this method, especially in terms of bias.

Even though RKHS, SVR and RF figured among the best methods in some of the situations analyzed, some of their tuning parameters were not optimized for each trait, due to the additional computational effort that would be required by this task, in a way that their performance might still be improved.

According to [33], predictive ability (or accuracy) is currently the main statistic employed to compare genomic prediction methods. However, bias and inflation of genomic predictions should be matter of concern, especially if animals from different generations and with different amounts of information (e.g. progeny-tested and newborn animals) are among selection candidates. If predictions are biased upwards, genetic trend will be overestimated, benefiting newborn animals unduly, while inflation would exaggerate differences among predicted values compared to the true differences, also having negative impact in selection schemes. Judged by their overall performance in terms of bias and inflation, RF, RKHS, SVR and RR_GBLUP outperformed the remainder methods.

It is also important to mention that in the present study the phenotypes used in model training were pre-corrected for environmental effects in order to reduce computing times, as well as the target phenotypes in the validation sets, what is not an optimal approach from a statistical perspective and could be an additional source of noise in the present results. For instance, in the case of overcorrection for some fixed effect in the validation set, it would be expected that methods that lead to more shrunk estimates of marker effects perform better in terms of bias and inflation in this situation.

Specifically with respect to SVR, this method had the lower overall accuracy among the ten methods studied by [5] when considering eight different datasets of crop species, even after optimizing the model for each trait. In present study, SVR figured among the best methods for W6W and WGS, and had very similar predictive performance to RKHS, what can be explained by the similar kernel definition in both methods. One possible explanation for the worse performance of SVR in [5] could be the fact that SVR was fitted using a linear kernel while a radial basis kernel was employed in this study.

It has been suggested that the accuracy of BayesCpi is not strongly affected by the starting value of π [19] and even by the lack of convergence in this parameter [34], although our results may suggest the need for further investigation of the impact of these factors with regard to bias and inflation of genomic predictions. An additional set of analyses was carried out to investigate the issue of convergence in π, by simulating two independent chains with different starting values for this parameter (0.90 and 0.10) and using the Gelman and Rubin's convergence test. The results varied across traits and also across replicates (data not shown), being that most of the replicates did not converge in the case of W6W and BL. For all traits, the averages of the posterior means of π across replicates were not strongly affected by the starting values, although the considerable variation in the estimates across replicates may suggest lack of information in the data to estimate π properly. Also, the poorer results with Bayes C could be justified by the misspecification of the value for π.

Heslot et al. [5] also pointed out that BayesCpi should not be recommended for application in genomic selection because it achieved very similar predictive ability to that of RR_GBLUP at a much greater computational cost. Conversely, BayesCpi presented some advantage over RR_GBLUP in the case of %CD8+ in the present study as well as in two of the 17 traits analyzed by [30]. Such disagreement demonstrates how difficult is to take a broad view on the relative performance of different methods and reinforces the hypothesis of interplay between relative performance of methods and genetic background.

The optimal method for genomic selection should be reliable across traits and computationally efficient, besides, obviously, being the highest accurate possible and less prone to overfitting [5]. Some authors have also alerted to the fact that methods relying more on LD between markers and QTL would be preferable to those whose accuracy result basically from the genetic relationships captured by the markers [27], because in this last case the accuracies are expected to decrease considerably in generations subsequent to estimation of marker effects. On the other hand, in situations of continuous updating of training populations and re-estimation of prediction equations, as typically occurs in dairy cattle (e.g. [35]), this not seems to be a major issue.

Given the imminent increase in dimensionality of genomic selection problems, due to both increase in the number of genotyped animals and especially in the density of marker panels [25], it is mandatory to take computing requirements in consideration when comparing statistical methods. Judged by their overall performance across traits and computational requirements, RR_GBLUP, RKHS and SVR seem to be particularly appealing. Although these methods have some conceptual differences, in both of them, problem dimensionality is reduced to the number of genotyped animals, what gives significant computational advantage over other methods when p >> n.

### Genetic architecture and predictive performance

It seems to be consensual that accuracy of genomic predictions is dependent on the genetic architecture of traits (number of underlying QTL, mode of inheritance), as well as on the size of the training set, the number of independent chromosome segments (which is function of genome size and effective population size), the heritability of (pseudo)phenotypes used to train models and the marker density (e.g. [3,25]).

Regarding to the relative performance of the prediction methods, Daetwyler et al. [3] suggested that the accuracy of GBLUP is invariant to number of QTL affecting the trait (N_QTL_), while the accuracy of methods based on variable selection is expected to be greater than that of GBLUP when N_QTL_ is lower than the number of independent chromosome segments.

In the present study, the predictive abilities were considerably greater in the case of within-family predictions when compared to across-family predictions, what was also reported by [6] and [7]. Despite this, this superiority of predictive ability for within-family predictions varied markedly across traits and this pattern was consistent across methods, what could suggest that predictive abilities for some traits are more dependent on close relationships than the others, and possibly are under a more polygenic background (larger N_QTL_).

Another hypothesis for the differences in the superiority of within-family predictions across traits, also mentioned by [7], is that resemblance between relatives, due to shared common environment (not properly accounted for in the model), could inflate predictive abilities and thus traits more influenced by such common environmental effects would exhibit larger superiority for within-family over across-family prediction.

Especially for W6W, the relatively good performance of POL and the different pattern for the of distribution of estimated marker effects in variable selection methods could be indicative of a more polygenic background than for the other traits.

In the other extreme, based on the previous results regarding QTL mapping of these traits [10] and given the superior predictive ability of variable selection methods for %CD8+ and CD4+/CD8+, one could expect that these traits are mostly influenced by a smaller number of QTL. In addition, there is some previous evidence that %CD8+ is influenced by dominance effects [7], although the two most accurate methods for this trait (LASSO and RF) do not explicitly take such effects into account.

It has been advocated RKHS, SVR and RF are able to capture complex interactions (e.g. dominance and epistatic effects), in a way that could be hypothesized that part of their predictive ability could be due to such interactions, although it can be difficult to confirm this theory, given the difficulty to model interactions explicitly. It is worth to emphasize that in this case these methods predict genotypic values rather than purely additive breeding values.

Within-family predictions, as defined in this study, are more similar to the situation expected in most of animal breeding applications [6], because selection candidates are expected to be related at some degree with animals of reference populations.

Another important question regarding to the predictive ability of genomic prediction methods regards to its variation across subpopulations or families. In the present study, there was some evidence that such differences in predictive ability are trait-specific, being that for the two traits for which suggestive differences between groups in predictive ability were found under some methods, phenotypic variances also differed between these groups. Thus, conversely to what was pointed out by [5], there was no evidence in the present study to reject the hypothesis that differences in the within-group predictive ability could be explained by differences in phenotypic variance.

According to [5], groups with larger phenotypic variance would have larger genetic variance and larger influence in model training, what would lead to higher predictive ability within these groups. Also, a possible explanation for such differences could be sampling, since predictive ability is expected to be higher for groups with larger number of individuals in the training set, although the use of 10 replicates is expected to ensure that all groups were properly represented in training set. In this way, this question still deserve further investigation in order to confirm the existence of such differences and to investigate their origin.

Some authors argue that genotyping by whole-genome resequencing will become a regular practice in the near future [25,36]. As the number of SNPs increases, the assumption that many of them do not have effect on a trait is more likely to be true [37], what would be a typical scenario in which variable selection methods would be preferable. Despite this, the first studies on analyses of full sequences did not signalize advantage of BayesB over GBLUP [25], although more research is needed to answer this question properly. For these reasons, comprehensive comparisons of statistical methods are still appealing in genomic selection, while further developments in terms of computational efficiency will be required to deal with problems of increasing dimensionality and complexity. More powerful tools will be probably needed to extend current models to account for pleiotropy (multi-trait predictions), non-additive genetic effects and genotype-by-environment interactions.

## Conclusions

Methods with large conceptual differences reached very similar predictive abilities in some situations and a clear re-ranking of methods was observed in function of the trait analyzed. For all traits and situations analyzed, at least two of the genomic prediction methods lead to more accurate predictions than the polygenic model.

Variable selection methods were more accurate than the remainder in the case of %CD8+ and CD4+/CD8+ and these traits are likely to be influenced by a smaller number of QTL than the remainder. Judged by their overall performance across traits and computational requirements, RR_GBLUP, RKHS and SVR are particularly appealing for application in genomic selection.

## Competing interests

The authors declare that they have no competing interests.

## Authors’ contributions

HHRN participated in the design of the study, carried out data analysis, participated in analysis of results and drafted the manuscript. RC participated in the design of the study, analysis of results and drafted the manuscript. SAQ participated in the design of the study and analysis of results and drafted the manuscript. All authors read and approved the final manuscript.
